# Antepartum Psychosis in the Setting of Preeclampsia With Severe Features: A Case Report

**DOI:** 10.7759/cureus.49678

**Published:** 2023-11-29

**Authors:** Matthan Moy, Anh Truonghuynh, Elvia Villarreal, Derek Neal

**Affiliations:** 1 Department of Psychiatry, University of Texas Medical Branch at Galveston, Galveston, USA; 2 Department of Obstetrics and Gynecology, University of Texas Medical Branch at Galveston, Galveston, USA

**Keywords:** women's mental health, substance use disorder, preeclampsia, antepartum psychosis, perinatal mental illness

## Abstract

The peripartum period, which can be further delineated into postpartum and antepartum periods, poses a heightened risk for psychiatric symptoms. While much is known about the risk of psychiatric symptoms in the postpartum period, antepartum psychosis remains exceedingly rare and poorly understood. This report presents a unique case of a 34-year-old pregnant woman with a past psychiatric history significant for recurrent major depressive disorder, generalized anxiety disorder, and substance use disorder of alcohol and marijuana, who developed preeclampsia with severe features and acute psychosis during the antepartum period. We explore the contributing factors to her presentation and discuss how each might have played a role in her symptoms. Although research exists on the connection between mood disorders and hypertensive disorders of pregnancy, studies that address the relationship between psychosis and hypertensive pregnancies are limited. Overall, the potential link between hypertensive disorders in pregnancy and peripartum psychosis has limited research and warrants further study.

## Introduction

Recent studies indicate a prevalence of perinatal psychiatric symptoms ranging from 14% to 30%, with mental disorders linked to an increased risk of pregnancy complications, including spontaneous abortion, preterm delivery, and hypertension [1]. Furthermore, infants born to mothers experiencing psychiatric symptoms during pregnancy may face potential risks such as low birth weight, gestational hypertension, perinatal death, and congenital malformations [2]. Notably, a study observed that women with postpartum psychiatric disorders had a higher mortality rate ratio than those without psychiatric history, with 40.6% of fatalities in this high-risk population attributed to unnatural causes such as suicides, accidents, and homicides, highlighting a significant increase in suicide risk [3].

Pregnant women not only face an elevated risk of psychiatric symptoms during or after gestation but are also highly susceptible to physiological disorders associated with pregnancy [4]. One significant obstetric complication is preeclampsia, characterized as a multisystem progressive disorder occurring after 20 weeks gestation, featuring new-onset hypertension with proteinuria or evidence of end-organ dysfunction. Preeclampsia, like many obstetric disorders, exhibits a spectrum of clinical features ranging from mild to severe cases. Severity determination involves parameters such as clinical signs (new-onset headache, vision changes, right upper quadrant pain) or lab values, including elevated liver enzymes [5]. Given its prevalence (approximately 5% of all pregnancies in the United States) and heightened risk of maternal/fetal complications, preeclampsia attracts significant interest for better understanding its etiology and treatment [6]. Moreover, the association between preeclampsia and maternal mental health often goes unnoticed. Recent studies reveal an increased risk and severity of psychiatric disorders in pregnant women who experience a hypertensive disorder during pregnancy, with many cases occurring immediately postpartum. Commonly reported psychiatric disorders in the study included depression, anxiety, and post-traumatic stress disorder (PTSD) [7].

This case reveals a rare presentation of an acutely psychotic episode experienced during the antepartum period in the setting of preeclampsia with severe features. Most, if not all, cases of pregnancy-related psychosis are experienced in the postpartum period occurring within the first six weeks after delivery [8], making the timing of this patient’s psychosis highly unusual.

## Case presentation

A 34-year-old woman at 38 weeks gestational age (G7P4034), with a history of recurrent major depressive disorder, generalized anxiety disorder, self-reported trauma related to an abusive spouse and a car accident, insomnia, panic disorder, and substance use disorder (particularly alcohol and marijuana), was brought by emergency medical services (EMS) for evaluation due to bizarre behavior. The call originated from her boyfriend, reporting increasingly odd behavior, including not sleeping for the past 24-48 hours, isolating herself in a closet for five hours, flat affect, and excessively calling her father (20-40 times) without speaking when he answered. Upon arrival at the emergency department (ED), the patient was conscious and alert but intermittently combative, attempting to bite and grab providers. Due to the severity of the behavior, the patient was placed in leather restraints. She was unable to provide a history. Vital signs revealed elevated blood pressure, with the highest recorded level at 151/92 mmHg. Suspecting preeclampsia, labs were ordered, showing unremarkable results except for elevated liver function enzymes, with alanine transaminase (ALT) at 115 U/L and aspartate aminotransferase (AST) at 155 U/L. A urine drug screen (UDS) was unable to be performed at the time, but a serum drug screen was taken and pending. 

The psychiatry team was consulted at the time with the patient exhibiting increasingly agitated behavior. At the outset of the interview, without any prompt, the patient stated “I love you too” and did not elaborate further when questioned. Throughout the interview, the patient exhibited word salad, inconsistent eye contact, and inability to directly answer the majority of questions posed. Language used by the patient included mostly non-sequiturs, curse words, and sexual themes. No paranoid themes were exhibited, and she was oriented to self but not to place, time, or situation. Collateral history from her boyfriend revealed that this unusual behavior may have been correlated with discussions regarding the paternity of their child that happened in the days leading up to her hospitalization. Furthermore, per her parents, she had no history of suicide attempts or past psychiatric hospitalizations. However, she had been seeing a psychiatrist since 2015 for long-standing major depressive disorder and generalized anxiety disorder with her last appointment having occurred two years prior. Her current medications included sertraline 50 mg daily and trazodone 50 mg as needed, although the level of adherence was unable to be determined. Further review of the patient chart revealed a strong family history of psychiatric disorders including mood disorders and substance use disorders. In addition, the patient had no history of hypertensive disorders in previous pregnancies. Recommendations included administering 5 mg haloperidol intramuscularly every six hours for 24 hours to address agitation. Additionally, 2 mg lorazepam was advised every four to six hours as needed for severe agitation or aggression, and 50 mg diphenhydramine every four to six hours as needed for anxiety or extrapyramidal symptoms. The patient was also recommended a 1:1 sitter for safety.

Before EMS transfer to the main hospital the following day, the patient was given a total of 12 mg haloperidol due to erratic behaviors in the morning consisting of attempting to dive off her hospital bed, spontaneously voiding, verbal aggression, and ripping out her IV. The patient was then switched from leather restraints to soft restraints during EMS transfer. Upon arrival at the main hospital, the patient was conscious and alert but not answering questions when prompted. Further labs revealed mildly elevated uric acid 7.3 mg/dL, lactate dehydrogenase (LDH) 723 U/L, creatine kinase (CK) 793 U/L, and acetaminophen < 10 µg/mL. Hepatitis labs and serum drug screen were negative. An ethics committee was consulted due to the patient’s lack of decision-making capacity, and the patient's parents were identified as surrogate decision makers.

Due to a lack of clear causes for her bizarre behavior, a CT head scan was completed without evidence of acute intracranial injury. Later in the day, after the patient had received three scheduled doses of haloperidol and an injection of lorazepam overnight, she was found to be more coherent and able to answer questions. The patient was unable to recall events leading up to hospitalization and repeatedly denied any drug or alcohol usage during the course of the pregnancy. A Foley catheter was placed for UDS which resulted presumptively positive for delta-9-tetrahydrocannabinol (THC) and negative for barbiturates, benzodiazepines, and tricyclic antidepressants. Repeat labs revealed stabilization of liver function enzymes with peak levels at AST 214 U/L and ALT 138 U/L, and a protein-to-creatinine (P:C) ratio of 0.4 thereby establishing preeclampsia diagnosis. The patient was started on magnesium sulfate for presumed HELLP (hemolysis, elevated liver enzymes, and low platelets) syndrome. Blood pressure was normalized with intermittently, mildly elevated ranges. The patient denied experiencing any symptoms of preeclampsia.

On day 3 of hospitalization, after the patient stabilized, she was induced and had a spontaneous vaginal delivery with no complications. The final diagnosis of preeclampsia with severe features was given in the setting of elevated blood pressures, P:C ratio > 0.3, and liver function tests greater than two times the upper limit of normal. Due to positive UDS, diagnosis of acute psychosis secondary to substance intoxication was determined as the most likely cause. Following delivery, Child Protective Services (CPS) was consulted and it was determined that the patient could be discharged once she was medically cleared; however, a live-in monitor was required for the following month. The patient followed up with the outpatient psychiatry clinic where she reported significant improvement in mood and was restarted on sertraline 50 mg daily with the addition of trazodone 25 mg as needed for insomnia.

## Discussion

There is extensive evidence in the literature that shows a correlation between the postpartum period and psychiatric disorders, which include but are not limited to subclinical symptoms such as postpartum blues or more severe cases of depression and psychosis. Most experts believe that these psychiatric symptoms are due to several biopsychosocial factors such as hormonal changes, physical exhaustion, and the major lifestyle change that comes with the new role of parenthood. Of these psychiatric disorders experienced in the postpartum period, psychosis has a rare occurrence of about one to two in every 1000 live births in the general population [9]. However, psychosis experienced in the antepartum period is exceedingly rare and not well documented within the literature. A literature search revealed a single case report from 2019 documenting a case of antepartum psychosis in a patient at 38 weeks gestation with no previous psychiatric disorders [10]. Comparatively, our patient has several risk factors worth exploring such as a previous psychiatric history, chronic substance abuse, and a hypertensive disorder of pregnancy. 

Given the unique circumstances surrounding the patient's admission, there are multiple factors that could potentially play a role in this patient’s presentation. One major risk factor that has been extensively demonstrated within literature is the pre-existence of psychiatric disorders. Our patient’s long-standing psychiatric history can be traced to 2015 and possibly even earlier given her self-reported history of abuse and trauma. Further risk factors include a strong family history of psychiatric disorders and social circumstances that may have exacerbated her high-stress state. However, her past psychiatric history is only one contributing factor that helps explain the psychotic symptoms experienced by the patient, which included physical and verbal aggression, disorganized behavior, disorientation, and a catatonic-like state. One particular organic cause in the patient’s case is substance intoxication as demonstrated through the patient’s positive urine drug screen for THC. Substance-induced psychosis is well-documented with one study suggesting an incidence of cannabis-induced intoxication ranging from 0.87% to 10.60% [11].

The current patient did not present with typical findings of acute marijuana intoxication. Additionally, family members noted her behavior to be unlike anything they had ever witnessed despite her long-standing use of marijuana. However, one possible explanation could be that the patient’s chronic usage of marijuana could have increased her likelihood of experiencing these symptoms. In fact, there has been recent research reporting psychosis as a possible side-effect in those who chronically use high-potency marijuana [12]. Regardless, the patient’s usage of substances during pregnancy highlights the need for adequate screening and counseling throughout the course of pregnancy. One study found that half of women with marijuana dependence will continue its usage during pregnancy [13].

One final risk factor that might explain the patient’s presentation is her preeclampsia with severe features. Given the acute onset of symptoms and temporal association with her preeclampsia, there is reason to believe that this hypertensive disorder may have also played a role in her bizarre presentation. This is also supported by her improvement on managing her preeclampsia and delivery. The pathophysiology of preeclampsia, which stems from immune-related abnormalities of the placenta that result in local and systemic anti-angiogenic and inflammatory factor production, gives reason to believe that this could have resulted in the patient’s psychotic symptomatology [14]. In fact, one study suggests an association between preeclampsia experienced during pregnancy and the incidence of postpartum psychiatric disorders due to the neurobiological impact of preeclampsia-related vascular pathology and inflammation; however, this study was limited in its findings regarding postpartum psychosis due to a limited number of cases, mainly representing unipolar depression and adjustment disorders [15]. Another study further demonstrated a relationship between hypertensive disorders of pregnancy and other psychiatric disorders but only reported findings on generalized anxiety disorder, depression, and post-traumatic stress disorder (PTSD) [16]. Because of the apparent lack of studies regarding hypertensive disorders of pregnancy and peripartum psychosis, further studies should be conducted to better understand the possible correlation that may exist between the two. By conducting more research regarding this correlation, better management and curated care can be given to expecting mothers during their pregnancies and postpartum.

## Conclusions

Overall, this report presents a rare case of antepartum psychosis in the setting of preeclampsia with severe features. We highlight the importance of managing perinatal psychosis given the heavy risk upon pregnant women. Although adequate research exists on the relationship between mood disorders and hypertensive disorders of pregnancy, there still remains a deficit in studies regarding the relationship between psychosis in pregnant women and hypertensive disorders of pregnancy, especially in the antepartum period. With more focused studies investigating the relationship between hypertensive disorders of pregnancy and psychosis, better management and care can be achieved for those afflicted.
